# The Impact of Automation and Digitalization in Hospital Medication Management: Economic Analysis in the European Countries

**DOI:** 10.3390/healthcare13131604

**Published:** 2025-07-04

**Authors:** Federico Filippo Orsini, Daniele Bellavia, Fabrizio Schettini, Emanuela Foglia

**Affiliations:** Healthcare Datascience LAB, LIUC—Università Carlo Cattaneo, 21053 Castellanza, Italy; forsini@liuc.it (F.F.O.); dbellavia@liuc.it (D.B.); efoglia@liuc.it (E.F.)

**Keywords:** hospital automation, hospital digitalization, economic impact, return on investment, net present value, payback time

## Abstract

Background/Objectives: European healthcare systems are increasingly adopting automation technologies to improve efficiency. This study evaluates the economic viability of hospital automation and medication management digitalization. Methods: An economic evaluation was based on a standardized hospital model comprising 561 beds, representative of an average acute care hospital across EU27 + UK. For each technology, several cost items were estimated using country-specific parameters such as labor costs, medication error rates, healthcare expenditure, and money discount rate. The financial metrics (Return On Investment—ROI, Net Present Value—NPV, Payback Time—PBT) were first calculated at the hospital level. These results were then extrapolated to the national level by scaling the per-hospital estimates according to the total number of hospital beds reported in each country. Finally, national results were aggregated to derive the overall European impact. Results: The analysis estimated a total European investment of EUR 3.55 billion, with an average PBT of 4.46 years and annual savings of 1,96 billion. ROI averaged 167%, and the total NPV was 8.21 billion. A major saving driver was the reduction in Medication Administration Errors that has an impact of 37.2% on the total savings. Payback times ranged from 3 years in high-GDP countries, to 7 years in lower-GDP nations. Conclusions: These findings demonstrate how providing structured data on hospital automation benefits could support decision-making processes, highlighting the organizational and economic feasibility of the investment across different European national contexts.

## 1. Introduction

In recent years, healthcare systems across Europe have increasingly focused their attention on automation and digitalization in medication management to enhance the efficiency and safety of hospital organizations processes. Despite this relevance recognition, specifically at hospital level, the current adoption of such technologies across the continent remains low, primarily due to high initial costs, the complexity of integration with existing hospital information and management systems, and a lack of clear, demonstrable Return On Investment (ROI) and feasibility evidence that could justify such fundings. These challenges have prevented widespread adoption, particularly in smaller healthcare facilities or in regions with limited budgets [1].

As of 17 January 2025, Regulation (EU) 2021/2282 on Health Technology Assessment (HTA) is fully applicable in all member states. This study fits into this renewed regulatory framework, contributing to the economic evidence needed for the technologies under assessment to be sustainable throughout their life cycle, including adoption in local settings.

This study aims to evaluate the economic viability and organizational impact of implementing medication management automation technologies in acute care hospitals across the 27 EU countries and the United Kingdom over a 10-year period (2024–2034). By modeling financial outcomes on a standardized hospital of 561 beds and scaling results according to national hospital bed capacity, the analysis provides aggregated estimates of investment costs, Payback Time, ROI, NPV, and annual savings at both national and European levels. The goal is to offer hospital administrators and policymakers a data-driven framework to assess the feasibility of automation investments based on hospital size and national healthcare context [2]. The studied technologies, optimizing the resources allocation, minimizing medication errors, and improving patients’ outcomes, could ensure the delivery of sustainable, high-quality care. The suggested shift toward digitalization and automation acknowledges the essential role of technology in enhancing healthcare standards and meeting future demands, despite current adoption barriers [3,4].

## 2. Materials and Methods

Following Bonnabry and François’s (2020) methodology [2], this study’s economic analysis is structured into three main stages: initial investment, annual cost–benefit balance, and calculation of ROI, NPV, and PBT.

ROI is a percentage measure of the profitability of an investment, indicating the efficiency of the capital employed.

$$ROI=\frac{\left(Total\;Costs-Total\;Savings\right)}{Total\;costs}$$

NPV evaluates the profitability of an investment by calculating the difference between the present values of cash inflows and outflows over the project’s duration (average European inflation rate 2.15%), with a positive NPV indicating that the project is expected to generate profit.

$$\sum_{t=2024}^{2034}\frac{Net\;Cash\;Flow}{({1+inflation\;rate)}^{t}}$$

PBT is used to determine the period necessary for the investment to recoup its initial costs through the cash flows it generates (in years), thanks to cost savings, providing a straightforward metric for financial planning in healthcare facilities.

This study assessed five automation technologies—inventory robots, unit dose systems, automated dispensing cabinets (ADCs), a central system in ICU for smart pumps, and oncology medication traceability—across the 27 EU countries and the United Kingdom over a projected 10-year period from 2024 to 2034. The economic data, updated with the average European inflation rate, supports uniformity and comparability across this timeframe, allowing a comprehensive evaluation of both the immediate and long-term economic and operational impacts of these technologies in hospital settings. The 10-year horizon is chosen based on the typical lifecycle of these technologies in healthcare, covering installation, full deployment, and mature operation, to assess their initial effects and sustained benefits comprehensively. This period helps ensure that the economic assessments capture the full spectrum of benefits and costs, providing a practical framework for decision-makers to evaluate the long-term impacts of healthcare automation investments.

The analysis was conducted using a baseline model of a medium-sized hospital with 561 beds, reflecting the structural capacity of a medium-sized healthcare organization across the EU27 + UK (Eurostat, 2024). For each automation technology, the required investment and the associated economic benefits were estimated at the hospital level, using country-specific input data (e.g., staff salaries, drug prices, average cost per hospital day) to enable the results’ contextualization and adaptation based on local healthcare structures and resources availability. Subsequently, to estimate the national-level impact, the results from the 561-bed hospital model were proportionally scaled up to the total number of care hospital beds in each country, based on the data reported by Eurostat (2024). This scaling approach provides a structured and conservative estimation of the potential economic impact of automation technologies at the national level, allowing for contextual adaptation across different healthcare systems. The assumptions and limitations of this method are addressed in the Section 4.

Subsequently, national estimates were adjusted to account for the current penetration rate of each technology in the different countries, based on data from the European Collaborative Action on Medication Errors and Traceability (ECAMET). This adjustment acknowledges that some hospitals have already implemented specific automation systems, and that technology adoption levels vary significantly across countries. As such, both the additional investment required and the achievable benefits were proportionally scaled according to existing adoption rates, ensuring more realistic and context-sensitive projections of economic impact.

To further enhance the accuracy of the model, country-specific adjustments were incorporated, including data on hospital bed capacity, average healthcare salaries (from SalaryExpert), ICU and non-ICU stay costs, and average drug prices. This combined approach ensures a balance between standardization and national-level specificity, supporting a fair and comprehensive evaluation of automation investments across diverse healthcare environments.

The results will be presented from two complementary perspectives: first, at the hospital level, illustrating the investment and savings associated with each technology in a modeled 561-bed European hospital; and second, at the aggregated level, summarizing the projected economic impact across the EU27 + UK. Additionally, country-specific results for all 28 nations included in the analysis are reported in the Appendixe A and Appendixe B to provide greater transparency, facilitating per country interpretation of the data.

Investment Calculation. The investment costs of the five automation technologies were estimated through an in-depth review of publicly available procurement documents and tender databases at the European level (e.g., TED—Tenders Electronic Daily). This approach ensured the use of real-world pricing information while maintaining neutrality and avoiding conflicts of interest [5]. For consistency and comparability, net prices were assumed to be constant across all EU27 + UK countries, while country-specific VAT rates were applied to reflect local fiscal frameworks. To support medication management and logistics processes in the reference hospital (561 beds), five automation technologies were considered, each targeting a specific phase of the medication-use cycle. These technologies were selected based on their relevance to clinical safety, operational efficiency, and prevalence in the extant literature.

- Inventory Management Robot

A robotic system installed in the central pharmacy to automate the storage, retrieval, and inventory control of medications. It uses barcode or RFID scanning to ensure accurate stock management, minimize picking errors, and optimize space utilization. It is designed to handle high volumes and improve traceability from the moment drugs enter the hospital supply chain.

- Unit Dose Distribution System (UDDS)

A centralized, automated packaging and labeling system that prepares individual unit doses for inpatients. It facilitates personalized and traceable drug dispensing at the patient level, reduces medication errors during preparation, and supports a closed-loop medication administration model.

- Automated Dispensing Cabinets (ADCs)

Eleven decentralized dispensing units located in clinical wards (e.g., medical, surgical, emergency departments) provide secure and on-demand access to medications. These systems are interfaced with the hospital information system and ensure controlled drug access, inventory tracking, and real-time documentation of drug dispensing activities.

- Centralized Dose Error Reduction System (DERS) for Smart Infusion Pumps in the ICU

A centralized digital library that standardizes infusion parameters (e.g., drug name, dose range, infusion rate) and programs smart pumps in critical care areas. It enables dose checking against predefined safety limits, reduces programming errors, and ensures consistent adherence to clinical protocols for high-risk intravenous therapies.

- Medication Traceability System for Oncologic Therapies

A dedicated platform that supports the safe prescription, preparation, and administration of chemotherapy drugs. It includes functionalities for computerized physician order entry (CPOE), gravimetric verification during compounding, automatic labeling, and digital documentation of each step. The system ensures full traceability of each dose, linking it to specific patients, operators, and preparation steps, and is specifically designed to address the complexity and risk profile of antineoplastic treatments.

Economic Benefits Classification. The economic benefits are systematically described and listed as follows.

- Reduction in Human Resource Costs: Automation technologies significantly reduce healthcare personnel time by eliminating or streamlining repetitive logistical tasks, such as drug picking, transportation, restocking, and manual documentation. To quantify the economic value of these time savings, we reviewed comparative studies from the literature reporting Full-Time Equivalent (FTE) workload differences between automated and non-automated hospital settings. Using these benchmarks, we estimated the annual workload (in hours per professional category) required to manage medication-related logistics in a 561-bed hospital without automation. These baseline values are reported in Table 1. For each automation technology, we applied corresponding efficiency improvement rates—also reported in Table 1—to estimate the reduction in workload. The time savings were monetized by multiplying the avoided hours by the average annual salary of the relevant personnel categories, using publicly available national wage data. This method was applied consistently across all technologies and countries, enabling a standardized estimation of human resource savings and a clearer understanding of how automation reallocates staff time from non-value-added tasks to clinical care [4].
- Reduction in Drug Wastage: Automated systems improve drug utilization by implementing stock rotation algorithms and real-time inventory visibility, which prioritize the dispensing and use of medications nearing expiration. This reduces the number of drug packages discarded due to expiry. Baseline wastage rates in non-automated settings and corresponding reduction percentages achievable with automation were obtained from published literature. For each drug class and automation technology, we estimated the annual volume of expired drugs, valued using the average purchase price per package. The difference in costs between the automated and non-automated scenarios was calculated as the economic benefit. All parameters used for this estimation—including baseline wastage rates, reduction percentages, and unit prices—are detailed in Table 1, along with the corresponding literature sources [6].
- Optimized Inventory Management: The optimization of inventory levels is increasingly seen as a critical goal for hospital logistics systems, especially in settings under economic pressure. Manual inventory management often leads to excess stock levels, especially of infrequently used pharmaceuticals, resulting in a high volume of capital tied up in unused inventory. Several studies have proposed predictive models to support hospital purchasing and supply planning activities, including approaches based on short time-series that have proven effective even in healthcare settings where long historical datasets are not always available [7]. Automation improves stock visibility, reorder accuracy, and rotation, thereby enabling hospitals to maintain optimal inventory levels. The economic benefit was estimated by comparing the average inventory value in automated versus non-automated settings, based on literature-derived benchmarks of stock reduction (expressed as a percentage of total drug value). Inventory holding costs were calculated by applying an annual holding cost rate—reflecting the opportunity cost of capital—sourced from the European Central Bank (ECB). All input parameters for this calculation, including baseline inventory levels, efficiency improvement rates, and holding cost percentage, are presented in Table 1, along with relevant references from the literature [8].
- Reduction in Medication Administration Errors (MAEs): Although the reduction in MAEs can be considered an indirect benefit, it was included in our analysis due to its significant clinical and economic implications, well documented in the literature (see references in Table 1). To estimate the effect of automation on MAEs, we relied on observational studies comparing automated and non-automated hospital workflows. For the first three technologies (inventory robots, Unit Dose Distribution Systems, and Automated Dispensing Cabinets), these studies provided error rates that we applied to a standardized 561-bed hospital model. For the remaining two technologies (Central DERS and Oncology Medication Traceability System), which primarily enhance error detection, we assumed an improvement in the recognition and correction of medication errors based on supportive evidence from the literature. We classified MAEs by severity—no harm, low harm, and mild/severe harm—and associated each category with an average increase in hospital length of stay, using values found in previous economic evaluations. To consolidate these values into a single figure, we calculated a weighted average increase in length of stay per error, based on the relative frequency of each severity category and its corresponding excess days of hospitalization. For each country, this average excess stay was multiplied by the mean inpatient cost per day, providing a country-specific estimate of the economic burden associated with a generic MAE. Automation-related savings were then derived by applying the expected reduction (or detection) rates to these cost estimates. Table 1 presents all input parameters used in this calculation, including baseline error rates, severity distribution, average LOS extension per error category, and unit costs across countries [9].

Table 1 details the parameters used to quantify both direct and indirect benefits for each automation technology examined. This format clearly delineates the specific impacts of deploying these technologies in hospital settings, emphasizing their contributions to operational efficiencies and cost reductions.

**Table 1:** Input parameters for economic evaluation of the technologies’ impact on an average European hospital.

Technology	Number of Medications Managed/Stocked in an Average European Hospital	Percentage of Expired or Wasted Drug/number. of Preparations	Percentage of Wasted Drugs’ Reduction Thanks to Automation	% Reduction in Product Stock Thanks to Automation	Number of Medications’ Errors Without Automation Technologies in a Year	Percentage of Medication Error Reduction Thanks to Automation	Professional	Number of Hours Dedicated to the Specific Activities Without Automation	Reduced Process Time Through Automation (% of hours)
Inventory Robots	1,563,133	0.46% [10]	−100.00% [11]	−26.40% [12]	128.96 [10]	−16% [10]	Technicians	7769.45 [10]	−31.40% [10]
Unit Dose System	545,143	0.55% [11]	−100.00% [11]	0.00% (Assumption)	77.79 [13]	−53% [13]	Nurses	20,640.59 [10]	−5.84% [14]
							Technicians	13,881.92 [10]	−10.00% [10]
Automated Dispensing Cabinets	2,850,352	0.55% [11]	−100.00% (Assumption)	−60.58% Elaboration from [12]	954.91 Elaboration from [15]	−53% [16]	Nurses	499.16 [10]	−80.00% [10]
							Pharmacists	1992.81 [10]	−50.00% [17]
							Technicians	1996.63 [10]	15.00% [18]
Smart pumps with DERS	26,690	-	-	-	12.82 Hypothesys: Baseline efficacy is equal to the one epressed in [19]	−100% (Assumption)	Nurses	394.37 [20]	−69.81% [20]
Med. Traceability System in Oncology	19,180	2.5153% (Elaboration from [21])	−100.00% (Assumption)	-	284.82 [22]	−100% (Assumption	Pharmacists (preparation)	2878.90 [23]	−44.38% [23]
		0.0960% [21]	−21.10% Elaboration from [21]		139.25 [24]	−75% [25,26,27]	Nurses (administration)	1917.99 [28]	−88.61% [28]

Summary Economic Metrics for Investment Evaluation: ROI, NPV over a 10-year horizon (country-specific discount rates, as published by the European Central Bank (ECB) in May 2024, were applied to ensure methodological consistency with national economic contexts), and PBT—were calculated for each country to provide a comprehensive economic assessment. Each metric provides unique insights into the financial viability of the investment, offering a holistic view of the automation economic impact in various contexts [29].

The model inputs, contents and economic results, were validated by five hospitals pharmacists, from different European Countries (two from Italy, and one each from France, Germany, and the UK). All participants had over ten years of experience in the hospital setting, with specific expertise in logistics and management. To ensure a structured validation process, the Nominal Group Technique (NGT) was implemented. This methodological approach facilitated the free expression of individual perspectives on critical and relevant elements, while subsequently fostering consensus around the findings to enhance their robustness. The CHEERS checklist was implemented to guarantee robustness of the study design.

Sensitivity Analysis: To assess the robustness of the economic model and incorporate contextual variability, a sensitivity analysis was conducted, focusing on two key parameters: hospital size and country-specific discount rates.

Regarding hospital size, the baseline scenario assumes a 561-bed facility, reflecting the average size of acute care hospitals across EU27 + UK (Eurostat, 2022). To simulate the influence of scale, the analysis includes two additional configurations, representing a smaller hospital with 449 beds and a larger one with 673 beds (±20%). Presenting results for hospitals of varying bed capacities equips European decision-makers with actionable insights tailored to their own facility size—whether larger or smaller than the 561-bed baseline—thereby enhancing the study’s generalizability and practical relevance for real-world investment decisions. The benefits associated with automation technologies were adjusted according to hospital size using a nonlinear coefficient β = 0.18. This coefficient was derived from comparative efficiency estimates reported in the OECD (2017) study “Tackwling Wasteful Spending on Health”, where observed savings in facilities of different sizes were used to estimate the elasticity of benefit with respect to volume. Specifically, by applying a log-transformation to the reported savings and hospital capacities, the resulting β-value captures how efficiency gains scale with hospital size. This allows the model to reflect economies of scale in a way that is consistent with real-world observations, ensuring that investment returns and operational savings vary realistically with institutional volume. The sensitivity analysis also includes national variability in discount rates, which directly affect Net Present Value (NPV) estimates.

Country-specific rates were collected from the European Central Bank (ECB, May 2024), and the model tested scenarios with ±20% deviations from each baseline rate to simulate potential macroeconomic shifts. This approach ensures that the financial outcomes remain robust across varying fiscal environments.

## 3. Results

Table 2 summarizes the average economic results for each technology at the hospital level, based on a 561-bed model. Findings account for country-specific variation in technology penetration, labor costs, and pharmaceutical pricing. Among the five technologies analyzed, the oncology medication traceability system demonstrates the most favorable economic profile, with the lowest average investment (EUR −78,549), high annual savings (EUR 106,777), and a Net Present Value (NPV) of EUR 656,242 over ten years—second only to the inventory robot. The inventory robot, while requiring a higher investment (EUR −190,163), yields the highest NPV overall (EUR 712,751), driven by substantial reductions in wastage and stock inefficiencies. Automated Dispensing Cabinets (ADCs), despite generating the highest annual savings (EUR 142,255), show a very low NPV (EUR 23,216) due to their significantly higher upfront costs (EUR −495,031). The unit-dose distribution system (UDDS) and DERS for ICU smart pumps occupy an intermediate position, combining moderate investment with solid savings and ROI profiles. These results highlight the importance of balancing investment requirements with achievable savings and suggest that technologies with strong safety and traceability components (such as oncology-focused systems) can offer substantial long-term value even with modest capital requirements.

To complement the hospital-level analysis, Table 3 presents the aggregated economic impact of implementing each technology across all acute care hospitals in the EU27 + UK. The estimates are based on standardized 561-bed hospital models, scaled by country-specific hospital counts and contextual variables (e.g., labor costs, drug prices, and penetration rates). Table 3 summarizes the key economic results per technology, highlighting their distinct profiles. The oncology medication traceability system emerged as the most economically favorable, with a 339% ROI, the shortest Payback Time (2 years), and substantial savings in both direct and indirect cost categories. Inventory robots also performed well, achieving a 232% ROI and over EUR 445 million in annual savings, primarily due to reductions in drug wastage and improved stock efficiency. Conversely, although Automated Dispensing Cabinets (ADCs) generated the highest total annual savings (EUR 556 million), their initial investment of EUR 1.76 billion resulted in a modest ROI of 14% and the longest Payback Time (8 years). These findings support differentiated investment strategies, depending on available capital, hospital size, and national healthcare cost structures, and offer policymakers a comparative overview of return potential across technologies at the system level.

The sensitivity analysis explored the effects of varying two structural assumptions: the size of the hospital infrastructure (±20% around the baseline of 561 beds) and the discount rate (±20%), which directly affects the Net Present Value (NPV) of the investments. Varying hospital size influenced the total economic returns at the European level, with the overall NPV ranging from approximately EUR 7.66 billion in the low-volume scenario to EUR 8.78 billion in the high-volume case. This confirmed the presence of economies of scale: larger infrastructures can dilute fixed investments more efficiently and generate proportionally higher returns. Nonetheless, ROI and Payback Time remained stable across scenarios, suggesting that the investment rationale holds even in smaller hospital settings. Varying the discount rate had a more selective impact. ROI and Payback Time remained unchanged, as they are not affected by discounting. However, NPV proved to be highly sensitive to this parameter, as expected. At the European level, reducing the discount rate by 20% increased the total NPV to approximately EUR 8.59 billion, while increasing it by 20% reduced NPV to EUR 7.86 billion, a difference of nearly EUR 730 million. Similar shifts were observed at the hospital level, although on a smaller scale. Across both sensitivity dimensions, the Medication Traceability System consistently delivered the strongest performance, maintaining high ROI and low Payback Time in all scenarios. Technologies with higher initial investments and longer return horizons—such as ACDs—were more exposed to variations in discount rate, with NPV differences of nearly EUR 160 million between scenarios. Table 4 resumes the main results for sensitivity analysis.

## 4. Discussion

The results of this study reinforce the economic viability of adopting automation and digital technologies in hospital medication management across Europe. With an estimated investment of EUR 3.55 billion and an average Payback Time (PBT) of 4.46 years, the financial sustainability of these technologies emerges clearly, especially in countries with high labor and drug costs. These findings are consistent with Bonnabry et al. (2022) [2], who estimated a PBT of 3.8 years for similar technologies in a Swiss context, thereby validating our approach and estimates across different settings.

Unlike previous studies focused on single countries or individual technologies, this research offers a comprehensive, cross-national economic evaluation analyzing EU27 + UK. By using a standardized hospital model and integrating country-specific parameters (e.g., wage levels, medication error rates, technology penetration), we provide robust estimates of Return on Investment (ROI), Net Present Value (NPV), and PBT both at the hospital and system level. This contributes new evidence to support strategic planning and investment decisions, especially in healthcare systems that are hesitant to invest in automation due to budget constraints or lack of detailed data on organizational benefits.

Notably, ROI and NPV remain positive even in lower-GDP countries, suggesting that automation is financially viable even in resource-constrained environments. The variation in ROI across nations highlights how contextual factors—such as workforce costs, existing infrastructure, and medication error prevalence—significantly influence the value generated from automation. These findings align with the literature emphasizing the importance of tailoring health technology investments to local economic and organizational contexts [30,31].

To improve the robustness of our estimates, we approached the sensitivity analysis by varying key parameters such as the number of hospital beds and the discount rate. Even under conservative assumptions (e.g., lower bed capacity or higher discount rates), the investment maintains a positive ROI and an acceptable PBT, confirming the model’s reliability across a range of economic scenarios.

In addition to economic benefits, automation technologies offer qualitative improvements—enhancing patient safety, reducing human error, and fostering more efficient workflows. These advantages are widely acknowledged in the literature [32] and are particularly relevant for reducing medication administration errors (MAEs), which represented the most significant driver of savings in our model.

However, several limitations must be acknowledged. First, our model assumes a standardized medication-use process across all countries, especially in oncology settings. Treatment protocols and workflows vary not only between countries, but often within regions. These variations can influence the actual impact of automation. Nonetheless, by focusing on core phases of medication management—prescription, preparation, dispensing, and administration—we ensure generalizability of results across care settings.

Second, the economic estimates rely on average values for variables such as labor costs, drug prices, and automation penetration, which introduces an inevitable approximation. Future research could explore hospital-level simulations using locally collected data to enhance precision.

We also recognize that the assumption of a 561-bed hospital may not fully reflect the heterogeneity of hospital sizes in Europe. In smaller hospitals, savings might be overestimated, while in larger facilities, economies of scale could yield even greater benefits. Nonetheless, this approach enables consistent and comparative analysis across countries and supports scalable policy recommendations. Furthermore, our two-step extrapolation—from hospital to national level, and then to EU27 + UK—ensures both micro- and macro-level insights.

Finally, it is worth noting that several countries have already implemented some of the evaluated technologies, creating asymmetries in current maturity and readiness. Countries such as Germany, France, and Sweden benefit from a stronger tradition of automation and thus serve as reference models. Conversely, countries newer to these technologies may face steeper learning curves and require phased implementation strategies, as previously discussed in the literature [33]. These differences, however, do not appear to significantly compromise the economic rationale for adoption.

Our analysis demonstrates the substantial economic and organizational value of investing in hospital automation. By combining empirical modeling with a sensitivity analysis and contextual interpretation, the study provides decision-makers with actionable evidence to guide sustainable innovation in healthcare.

## 5. Conclusions

In an era of constrained healthcare budgets, rigorous economic evaluations are crucial to justify investments in automation and digitalization. This study offers a structured and scalable methodology for calculating ROI, NPV, and Payback Time, equipping hospital pharmacists and decision-makers with essential tools to guide strategic investments in medication-use technologies.

Our findings confirm the economic sustainability of automation, with a total estimated investment of EUR 3.55 billion and an average Payback Time of 4.46 years across EU27 + UK. Even under varying economic assumptions—such as changes in hospital size or inflation rates—the model remains robust, highlighting the resilience of these investments over time and across contexts.

Beyond financial metrics, the study supports a broader vision of sustainable healthcare transformation. The ability to quantify both direct and indirect benefits enhances the case for adoption, particularly in systems where automation uptake has been limited by lack of economic evidence.

This methodology can be adapted to local contexts and evolving policy frameworks, offering a decision-support tool for national health authorities and hospital managers alike. Future research could refine the model using real-world data from diverse hospital settings, contributing to even more precise and contextualized investment planning.

## Figures and Tables

**Table 2:** Economic impact of automated technologies on an average European hospital.

Hospital-Based results			
Technology	Investment	Annual Savings	NPV
Inventory Robot	−190,163 EUR	140,488 EUR	780,692 EUR
UDDS	−151,171 EUR	55,301 EUR	186,550 EUR
ACDs	−495,031 EUR	151,310 EUR	96,363 EUR
DERS	−99,017 EUR	65,300 EUR	304,488 EUR
Med, Traceability System	−78,549 EUR	111,783 EUR	696,935 EUR
Total	−1,013,931 EUR	524,183 EUR	2,065,029 EUR

**Table 3:** Economic impact of automated technologies on all European Healthcare Services.

Technology	Investment	HR Efficiency Savings	Wastage Reduction Savings	Inventory Reduction Savings	MAE Reduction Savings (Indirect Benefit)	Total Annual Savings	ROI	NPV	Payback Time
Inventory Robot	−600,130,638 EUR	105,401,590 EUR	298,558,225 EUR	61,611,921 EUR	8,807,528 EUR	474,379,264 EUR	253%	2,871,105,529 EUR	2.75
UDDS	−507,585,002 EUR	100,732,484 EUR	98,798,224 EUR	0 EUR	14,016,421 EUR	213,547,129 EUR	88%	843,421,857 EUR	5.5
ACDs	−1,760,264,736 EUR	98,356,058 EUR	182,768,227 EUR	72,386,386 EUR	238,727,714 EUR	592,238,384 EUR	22%	887,897,647 EUR	7.33
DERS	−385,739,250 EUR	19,952,327 EUR	0 EUR	0 EUR	208,915,013 EUR	228,867,340 EUR	114%	1,031,345,807 EUR	3.75
Med, Traceability System	−304,110,137 EUR	162,230,625 EUR	36,029,794 EUR	0 EUR	262,662,674 EUR	460,923,093 EUR	360%	3,046,279,644 EUR	2.25
Total	−3,557,829,764 EUR	486,673,083 EUR	616,154,470 EUR	133,998,307 EUR	733,129,349 EUR	1,969,955,209 EUR	167%	8,213,739,492 EUR	4.46

**Table 4:** Main sensitivity analysis results.

Scenario	ROI	Total NPV (EUR)	Payback Time (Years)
Baseline	167%	EUR 8,213,739,492	4.46
Hospital size −20%	133%	EUR 7,661,066,552	4.71
Hospital size +20%	164%	EUR 8,778,311,040	4.93
Discount rate −20%	167%	EUR 8,585,215,964	4.39
Discount rate +20%	167%	EUR 7,859,352,036	4.46

## Data Availability

The original contributions presented in this study are included in the article. Further inquiries can be directed to the corresponding author.

## Abbreviations

The following abbreviations are used in this manuscript:

ROI	Return On Investment
NPV	Net Present Value
PBT	Payback Time
FTE	Full-Time Equivalent
UDDS	Unit Dose Dispensing System
ADCs	Automated Dispensing Cabinets
DERS	Dose Error Reduction System

## Appendix A

This section presents the results of the economic evaluation calculated at the national level, detailing the estimated impact of each of the five automation and digital technologies assessed in this study. The aim is to provide country-specific insights that may support policymakers and healthcare decision-makers across Europe in evaluating the potential benefits of investing in individual technologies, based on their local priorities and resource availability.

**Table A1:** Economic impact after the full implementation of inventory robot in all hospitals of each nation.

Country	Inventory Robot Investment	Inventory Robot HR Efficiency Savings	Inventory Robot Wastage Reduction Savings	Inventory Robot Inventory Reduction Savings	Inventory Robot MAE Reduction Savings (Indirect)	Inventory Robot Total Savings	Inventory Robot ROI	Inventory Robot NPV	Inventory Robot Payback Time
Austria	9,537,524 EUR	−2,677,887 EUR	−5,318,178 EUR	−1,097,485 EUR	−156,887 EUR	−9,250,437 EUR	320%	−56,236,844 EUR	2
Belgium	17,572,407 EUR	−4,763,417 EUR	−8,788,508 EUR	−1,813,639 EUR	−259,263 EUR	−15,624,827 EUR	276%	−87,730,558 EUR	2
Bulgaria	17,134,280 EUR	−830,112 EUR	−7,806,219 EUR	−1,610,929 EUR	−230,285 EUR	−10,477,546 EUR	173%	−55,876,212 EUR	3
Croatia	5,762,476 EUR	−405,847 EUR	−2,415,304 EUR	−498,434 EUR	−71,252 EUR	−3,390,837 EUR	155%	−16,434,051 EUR	3
Cyprus	1,059,581 EUR	−85,896 EUR	−506,289 EUR	−104,480 EUR	−14,936 EUR	−711,600 EUR	199%	−3,991,610 EUR	3
Czechia	9,518,597 EUR	−3,492,428 EUR	−5,188,877 EUR	−1,070,802 EUR	−153,073 EUR	−9,905,179 EUR	364%	−65,441,247 EUR	2
Denmark	3,551,425 EUR	−1,082,681 EUR	−1,870,432 EUR	−385,991 EUR	−55,178 EUR	−3,394,282 EUR	322%	−21,465,708 EUR	2
Estonia	2,290,903 EUR	−213,491 EUR	−809,371 EUR	−167,026 EUR	−23,877 EUR	−1,213,765 EUR	127%	−5,297,560 EUR	4
Finland	2,940,295 EUR	−776,859 EUR	−1,293,507 EUR	−266,934 EUR	−38,159 EUR	−2,375,458 EUR	263%	−14,741,125 EUR	3
France	116,594,957 EUR	−23,963,002 EUR	−55,160,788 EUR	−11,383,247 EUR	−1,627,254 EUR	−92,134,292 EUR	255%	−566,889,586 EUR	3
Germany	103,801,385 EUR	−23,641,149 EUR	−61,820,802 EUR	−12,757,640 EUR	−1,823,726 EUR	−100,043,318 EUR	322%	−622,429,538 EUR	2
Greece	15,334,439 EUR	−1,631,499 EUR	−7,031,650 EUR	−1,451,085 EUR	−207,435 EUR	−10,321,670 EUR	196%	−55,991,686 EUR	3
Hungary	16,463,752 EUR	−913,016 EUR	−6,792,002 EUR	−1,401,631 EUR	−200,365 EUR	−9,307,014 EUR	129%	−37,339,855 EUR	3
Ireland	5,411,715 EUR	−1,330,238 EUR	−3,315,354 EUR	−684,172 EUR	−97,804 EUR	−5,427,568 EUR	342%	−34,607,617 EUR	2
Italy	58,197,246 EUR	−10,936,904 EUR	−34,861,599 EUR	−7,194,208 EUR	−1,028,424 EUR	−54,021,136 EUR	304%	−327,920,917 EUR	2
Latvia	3,760,824 EUR	−288,731 EUR	−1,339,672 EUR	−276,461 EUR	−39,521 EUR	−1,944,385 EUR	116%	−7,881,271 EUR	4
Lithuania	5,163,697 EUR	−396,434 EUR	−1,839,400 EUR	−379,587 EUR	−54,263 EUR	−2,669,684 EUR	124%	−11,874,390 EUR	4
Luxembourg	741,385 EUR	−167,356 EUR	−383,466 EUR	−79,134 EUR	−11,312 EUR	−641,269 EUR	291%	−4,132,653 EUR	2
Malta	587,631 EUR	−88,685 EUR	−283,161 EUR	−58,435 EUR	−8,353 EUR	−438,634 EUR	231%	−2,549,685 EUR	3
Netherlands	15,980,608 EUR	−4,057,855 EUR	−8,210,314 EUR	−1,694,320 EUR	−242,206 EUR	−14,204,695 EUR	294%	−88,300,454 EUR	2
Poland	64,939,682 EUR	−4,966,744 EUR	−22,756,516 EUR	−4,696,145 EUR	−671,322 EUR	−33,090,726 EUR	106%	−120,556,135 EUR	4
Portugal	10,920,430 EUR	−1,563,248 EUR	−5,327,766 EUR	−1,099,464 EUR	−157,170 EUR	−8,147,647 EUR	229%	−46,720,000 EUR	3
Romania	40,134,571 EUR	−2,727,393 EUR	−17,670,319 EUR	−3,646,533 EUR	−521,278 EUR	−24,565,523 EUR	153%	−109,694,626 EUR	3
Slovakia	10,002,195 EUR	−1,048,285 EUR	−4,218,929 EUR	−870,639 EUR	−124,459 EUR	−6,262,312 EUR	161%	−28,773,102 EUR	3
Slovenia	3,505,086 EUR	−939,458 EUR	−1,505,260 EUR	−310,633 EUR	−44,405 EUR	−2,799,757 EUR	245%	−15,776,756 EUR	3
Spain	31,305,709 EUR	−5,206,229 EUR	−16,336,699 EUR	−3,371,320 EUR	−481,936 EUR	−25,396,184 EUR	249%	−143,074,795 EUR	3
Sweden	5,032,795 EUR	−1,690,249 EUR	−2,266,855 EUR	−467,799 EUR	−66,873 EUR	−4,491,776 EUR	291%	−27,252,913 EUR	2
Total EU27	577,245,596 EUR	−99,885,092 EUR	−285,117,237 EUR	−58,838,174 EUR	−8,411,016 EUR	−452,251,520 EUR	250%	−2,578,980,894 EUR	3
United Kingdom	22,885,042 EUR	−5,516,497 EUR	−13,440,988 EUR	−2,773,747 EUR	−396,512 EUR	−22,127,744 EUR	329%	−141,329,149 EUR	2
Total EU27 + UK	600,130,638 EUR	−105,401,590 EUR	−298,558,225 EUR	−61,611,921 EUR	−8,807,528 EUR	−474,379,264 EUR	253%	−2,720,310,043 EUR	3

**Table A2:** Economic impact after the full implementation of UDDS in all hospitals of each nation.

Country	Unit Dose System Investment	Unit Dose System HR Efficiency Savings	Unit Dose System Wastage Reduction Savings	Unit Dose System Inventory Reduction Savings	Unit Dose System MAE Reduction Savings (Indirect)	Unit Dose System Total Savings	Unit Dose System ROI	Unit Dose System NPV	Unit Dose System Payback Time
Austria	15,245,165 EUR	−4,751,008 EUR	−3,178,521 EUR	0 EUR	−450,934 EUR	−8,380,463 EUR	138%	−38,766,533 EUR	3
Belgium	2,440,612 EUR	−711,227 EUR	−456,403 EUR	0 EUR	−64,749 EUR	−1,232,380 EUR	113%	−5,009,576 EUR	4
Bulgaria	19,038,089 EUR	−1,133,957 EUR	−3,243,127 EUR	0 EUR	−460,100 EUR	−4,837,184 EUR	13%	−4,777,031 EUR	8
Croatia	6,402,752 EUR	−546,183 EUR	−1,003,449 EUR	0 EUR	−142,358 EUR	−1,691,990 EUR	14%	−1,694,651 EUR	8
Cyprus	828,924 EUR	−72,944 EUR	−148,096 EUR	0 EUR	−21,010 EUR	−242,050 EUR	30%	−472,765 EUR	7
Czechia	15,214,912 EUR	−3,928,253 EUR	−3,101,241 EUR	0 EUR	−439,971 EUR	−7,469,465 EUR	119%	−34,167,764 EUR	4
Denmark	3,946,027 EUR	−1,314,620 EUR	−777,079 EUR	0 EUR	−110,244 EUR	−2,201,942 EUR	147%	−10,845,899 EUR	3
Estonia	1,527,269 EUR	−163,943 EUR	−201,754 EUR	0 EUR	−28,623 EUR	−394,320 EUR	10%	−290,087 EUR	9
Finland	3,266,994 EUR	−899,022 EUR	−537,393 EUR	0 EUR	−76,239 EUR	−1,512,654 EUR	108%	−6,732,907 EUR	4
France	31,157,584 EUR	−7,201,779 EUR	−5,511,632 EUR	0 EUR	−781,931 EUR	−13,495,342 EUR	95%	−56,242,260 EUR	4
Germany	165,920,343 EUR	−42,755,678 EUR	−36,948,506 EUR	0 EUR	−5,241,853 EUR	−84,946,037 EUR	124%	−383,683,750 EUR	4
Greece	11,996,330 EUR	−1,736,215 EUR	−2,056,854 EUR	0 EUR	−291,804 EUR	−4,084,873 EUR	50%	−11,096,804 EUR	6
Hungary	18,293,058 EUR	−1,388,291 EUR	−2,821,767 EUR	0 EUR	−400,322 EUR	−4,610,379 EUR	2%	−681,393 EUR	10
Ireland	6,013,017 EUR	−1,550,142 EUR	−1,377,378 EUR	0 EUR	−195,407 EUR	−3,122,927 EUR	129%	−14,489,059 EUR	4
Italy	45,528,458 EUR	−9,601,665 EUR	−10,197,496 EUR	0 EUR	−1,446,710 EUR	−21,245,872 EUR	103%	−87,014,711 EUR	4
Latvia	2,507,216 EUR	−155,982 EUR	−333,943 EUR	0 EUR	−47,376 EUR	−537,301 EUR	−10%	463,170 EUR	Payback time is over 10 years
Lithuania	3,442,464 EUR	−214,805 EUR	−458,512 EUR	0 EUR	−65,049 EUR	−738,365 EUR	−7%	439,693 EUR	Payback time is over 10 years
Luxembourg	0 EUR	0 EUR	0 EUR	0 EUR	0 EUR	0 EUR	Penetration rate 100%	Penetration rate 100%	Penetration rate 100%
Malta	459,711 EUR	−81,305 EUR	−82,829 EUR	0 EUR	−11,751 EUR	−175,885 EUR	70%	−601,161 EUR	5
Netherlands	0 EUR	0 EUR	0 EUR	0 EUR	0 EUR	0 EUR	Penetration rate 100%	Penetration rate 100%	Penetration rate 100%
Poland	43,293,122 EUR	−4,045,104 EUR	−5,672,575 EUR	0 EUR	−804,763 EUR	−10,522,442 EUR	−2%	1,375,267 EUR	Payback time is over 10 years
Portugal	1,198,401 EUR	−175,075 EUR	−218,612 EUR	0 EUR	−31,014 EUR	−424,700 EUR	56%	−1,258,320 EUR	5
Romania	44,593,967 EUR	−3,111,871 EUR	−7,341,210 EUR	0 EUR	−1,041,491 EUR	−11,494,572 EUR	7%	−5,286,506 EUR	9
Slovakia	11,113,550 EUR	−1,477,387 EUR	−1,752,772 EUR	0 EUR	−248,664 EUR	−3,478,824 EUR	30%	−6,029,583 EUR	7
Slovenia	3,894,540 EUR	−725,357 EUR	−625,367 EUR	0 EUR	−88,720 EUR	−1,439,444 EUR	59%	−4,260,977 EUR	5
Spain	0 EUR	0 EUR	0 EUR	0 EUR	0 EUR	0 EUR	Penetration rate 100%	Penetration rate 100%	Penetration rate 100%
Sweden	5,591,994 EUR	−1,538,227 EUR	−941,775 EUR	0 EUR	−133,609 EUR	−2,613,610 EUR	105%	−10,898,491 EUR	4
Total EU27	462,914,499 EUR	−89,280,038 EUR	−88,988,291 EUR	0 EUR	−12,624,694 EUR	−190,893,022 EUR	84%	−682,022,097 EUR	5
United Kingdom	44,670,503 EUR	−11,452,446 EUR	−9,809,933 EUR	0 EUR	−1,391,727 EUR	−22,654,107 EUR	125%	−104,763,161 EUR	4
Total EU27 + UK	507,585,002 EUR	−100,732,484 EUR	−98,798,224 EUR	0 EUR	−14,016,421 EUR	−213,547,129 EUR	88%	−786,785,258 EUR	4

**Table A3:** Economic impact after the full implementation of ADCs in all hospitals of each nation.

Country	Automated Dispensing Cabinets Investment	Automated Dispensing Cabinets HR Efficiency Savings	Automated Dispensing Cabinets Wastage Reduction Savings	Automated Dispensing Cabinets Inventory Reduction Savings	Automated Dispensing Cabinets MAE Reduction Savings (Indirect)	Automated Dispensing Cabinets Total Savings	ADC ROI	ADC NPV	ADC Payback Time
Austria	42,477,016 EUR	−3,330,725 EUR	−4,771,350 EUR	−1,889,720 EUR	−6,232,230 EUR	−16,224,025 EUR	34%	−33,206,716 EUR	6
Belgium	32,090,235 EUR	−2,396,951 EUR	−3,233,084 EUR	−1,280,481 EUR	−4,222,980 EUR	−11,133,496 EUR	20%	−14,057,340 EUR	7
Bulgaria	39,108,994 EUR	−1,084,398 EUR	−3,589,316 EUR	−1,421,569 EUR	−4,688,283 EUR	−10,783,566 EUR	0%	437,818 EUR	Payback time is over 10 years
Croatia	13,152,852 EUR	−341,090 EUR	−1,110,562 EUR	−439,844 EUR	−1,450,591 EUR	−3,342,087 EUR	−11%	3,138,680 EUR	Payback time is over 10 years
Cyprus	2,765,181 EUR	−75,748 EUR	−266,163 EUR	−105,415 EUR	−347,656 EUR	−794,983 EUR	4%	−243,065 EUR	10
Czechia	42,392,722 EUR	−1,074,645 EUR	−4,655,344 EUR	−1,843,775 EUR	−6,080,705 EUR	−13,654,469 EUR	16%	−16,078,638 EUR	8
Denmark	12,004,555 EUR	−986,488 EUR	−1,273,636 EUR	−504,431 EUR	−1,663,595 EUR	−4,428,151 EUR	32%	−8,927,853 EUR	6
Estonia	5,414,049 EUR	−179,318 EUR	−385,321 EUR	−152,609 EUR	−503,298 EUR	−1,220,546 EUR	−21%	2,596,971 EUR	Payback time is over 10 years
Finland	9,938,809 EUR	−729,463 EUR	−880,790 EUR	−348,842 EUR	−1,150,468 EUR	−3,109,561 EUR	14%	−3,220,562 EUR	8
France	265,082,878 EUR	−17,200,629 EUR	−25,263,440 EUR	−10,005,728 EUR	−32,998,533 EUR	−85,468,330 EUR	17%	−107,571,072 EUR	8
Germany	462,297,452 EUR	−33,049,912 EUR	−55,464,245 EUR	−21,966,927 EUR	−72,446,139 EUR	−182,927,223 EUR	41%	−431,561,390 EUR	6
Greece	39,587,888 EUR	−1,925,701 EUR	−3,656,889 EUR	−1,448,332 EUR	−4,776,546 EUR	−11,807,468 EUR	6%	−5,762,508 EUR	9
Hungary	37,578,515 EUR	−1,002,977 EUR	−3,122,977 EUR	−1,236,872 EUR	−4,079,161 EUR	−9,441,988 EUR	−16%	13,036,748 EUR	Payback time is over 10 years
Ireland	11,161,662 EUR	−888,126 EUR	−1,377,476 EUR	−545,557 EUR	−1,799,228 EUR	−4,610,386 EUR	48%	−12,257,144 EUR	5
Italy	150,243,911 EUR	−8,405,259 EUR	−18,130,170 EUR	−7,180,556 EUR	−23,681,217 EUR	−57,397,201 EUR	35%	−120,509,066 EUR	6
Latvia	8,887,886 EUR	−186,043 EUR	−637,784 EUR	−252,598 EUR	−833,060 EUR	−1,909,485 EUR	−26%	5,156,987 EUR	Payback time is over 10 years
Lithuania	12,203,267 EUR	−255,744 EUR	−875,692 EUR	−346,823 EUR	−1,143,810 EUR	−2,622,069 EUR	−24%	6,699,946 EUR	Payback time is over 10 years
Luxembourg	1,834,928 EUR	−211,861 EUR	−191,189 EUR	−75,721 EUR	−249,727 EUR	−728,498 EUR	45%	−1,959,036 EUR	6
Malta	1,517,048 EUR	−82,032 EUR	−147,261 EUR	−58,324 EUR	−192,349 EUR	−479,966 EUR	14%	−479,647 EUR	8
Netherlands	39,552,005 EUR	−2,923,760 EUR	−4,093,503 EUR	−1,621,255 EUR	−5,346,840 EUR	−13,985,357 EUR	27%	−24,767,037 EUR	7
Poland	153,470,733 EUR	−4,622,264 EUR	−10,833,812 EUR	−4,290,792 EUR	−14,150,880 EUR	−33,897,748 EUR	−27%	86,828,881 EUR	Payback time is over 10 years
Portugal	29,220,155 EUR	−1,118,407 EUR	−2,871,759 EUR	−1,137,376 EUR	−3,751,027 EUR	−8,878,570 EUR	9%	−5,789,569 EUR	9
Romania	91,607,157 EUR	−2,202,736 EUR	−8,124,849 EUR	−3,217,892 EUR	−10,612,493 EUR	−24,157,970 EUR	−11%	21,005,960 EUR	Payback time is over 10 years
Slovakia	22,830,010 EUR	−881,563 EUR	−1,939,872 EUR	−768,297 EUR	−2,533,817 EUR	−6,123,550 EUR	−9%	4,271,938 EUR	Payback time is over 10 years
Slovenia	8,000,360 EUR	−167,032 EUR	−692,122 EUR	−274,119 EUR	−904,034 EUR	−2,037,306 EUR	−11%	1,908,494 EUR	Payback time is over 10 years
Spain	81,263,924 EUR	−4,416,519 EUR	−8,542,769 EUR	−3,383,412 EUR	−11,158,371 EUR	−27,501,071 EUR	19%	−34,016,945 EUR	700%
Sweden	17,011,895 EUR	−770,674 EUR	−1,543,574 EUR	−611,341 EUR	−2,016,181 EUR	−4,941,770 EUR	3%	−1,288,881 EUR	1000%
Total EU27	1,632,696,089 EUR	−90,510,064 EUR	−167,674,950 EUR	−66,408,609 EUR	−219,013,218 EUR	−543,606,841 EUR	20%	−676,614,045 EUR	7
United Kingdom	127,568,647 EUR	−7,845,993 EUR	−15,093,277 EUR	−5,977,777 EUR	−19,714,496 EUR	−48,631,543 EUR	37%	−109,240,844 EUR	600%
Total EU27 + UK	1,760,264,736 EUR	−98,356,058 EUR	−182,768,227 EUR	−72,386,386 EUR	−238,727,714 EUR	−592,238,384 EUR	22%	−785,854,889 EUR	7

**Table A4:** Economic impact after the full implementation of DERS in all hospitals of each nation.

Country	DERS Investment	DERS HR Efficiency Savings	DERS Wastage Reduction Savings	DERS Inventory Reduction Savings	DERS MAE Reduction Savings (Indirect)	DERS Total Savings	DERS ROI	DERS NPV	DERS Payback Time
Austria	8,831,041 EUR	−676,837 EUR	0 EUR	0 EUR	−6,300,363 EUR	−6,977,200 EUR	178%	−35,668,813 EUR	3
Belgium	10,982,754 EUR	−765,228 EUR	0 EUR	0 EUR	−8,215,731 EUR	−8,980,959 EUR	182%	−44,374,438 EUR	3
Bulgaria	7,900,807 EUR	−125,371 EUR	0 EUR	0 EUR	−3,454,788 EUR	−3,580,159 EUR	64%	−11,716,313 EUR	5
Croatia	2,657,142 EUR	−59,738 EUR	0 EUR	0 EUR	−1,068,938 EUR	−1,128,676 EUR	50%	−2,985,356 EUR	5
Cyprus	486,542 EUR	−10,327 EUR	0 EUR	0 EUR	−275,397 EUR	−285,723 EUR	112%	−1,271,059 EUR	4
Czechia	8,813,516 EUR	−282,852 EUR	0 EUR	0 EUR	−3,951,482 EUR	−4,234,334 EUR	73%	−15,096,045 EUR	4
Denmark	1,973,014 EUR	−159,484 EUR	0 EUR	0 EUR	−2,589,230 EUR	−2,748,715 EUR	399%	−18,212,922 EUR	2
Estonia	763,634 EUR	−20,788 EUR	0 EUR	0 EUR	−314,756 EUR	−335,544 EUR	53%	−910,405 EUR	5
Finland	1,633,497 EUR	−104,330 EUR	0 EUR	0 EUR	−2,160,963 EUR	−2,265,293 EUR	404%	−15,547,293 EUR	2
France	76,254,086 EUR	−4,383,255 EUR	0 EUR	0 EUR	−41,315,860 EUR	−45,699,115 EUR	118%	−211,695,232 EUR	3
Germany	96,112,394 EUR	−6,190,202 EUR	0 EUR	0 EUR	−43,815,585 EUR	−50,005,786 EUR	85%	−187,357,789 EUR	4
Greece	7,041,324 EUR	−290,144 EUR	0 EUR	0 EUR	−3,824,878 EUR	−4,115,022 EUR	108%	−17,541,418 EUR	4
Hungary	7,591,619 EUR	−164,627 EUR	0 EUR	0 EUR	−3,005,928 EUR	−3,170,554 EUR	39%	−6,360,431 EUR	6
Ireland	3,006,508 EUR	−181,095 EUR	0 EUR	0 EUR	−2,677,561 EUR	−2,858,656 EUR	240%	−16,626,461 EUR	2
Italy	26,723,225 EUR	−1,399,008 EUR	0 EUR	0 EUR	−14,754,143 EUR	−16,153,151 EUR	114%	−69,383,017 EUR	3
Latvia	1,253,608 EUR	−13,106 EUR	0 EUR	0 EUR	−520,984 EUR	−534,090 EUR	46%	−1,267,132 EUR	5
Lithuania	1,721,232 EUR	−18,141 EUR	0 EUR	0 EUR	−715,323 EUR	−733,463 EUR	50%	−1,972,598 EUR	5
Luxembourg	411,881 EUR	−39,572 EUR	0 EUR	0 EUR	−318,644 EUR	−358,216 EUR	218%	−2,124,919 EUR	3
Malta	269,831 EUR	−12,268 EUR	0 EUR	0 EUR	−154,026 EUR	−166,294 EUR	121%	−759,500 EUR	3
Netherlands	8,878,116 EUR	−712,312 EUR	0 EUR	0 EUR	−4,702,174 EUR	−5,414,486 EUR	119%	−24,514,471 EUR	3
Poland	21,646,561 EUR	−536,383 EUR	0 EUR	0 EUR	−8,849,762 EUR	−9,386,146 EUR	44%	−20,378,915 EUR	5
Portugal	7,265,307 EUR	−241,446 EUR	0 EUR	0 EUR	−4,988,515 EUR	−5,229,961 EUR	157%	−26,348,568 EUR	3
Romania	18,506,496 EUR	−295,598 EUR	0 EUR	0 EUR	−7,820,330 EUR	−8,115,928 EUR	49%	−19,632,341 EUR	5
Slovakia	4,612,123 EUR	−166,924 EUR	0 EUR	0 EUR	−2,085,047 EUR	−2,251,971 EUR	66%	−6,703,674 EUR	5
Slovenia	1,616,234 EUR	−36,153 EUR	0 EUR	0 EUR	−666,181 EUR	−702,334 EUR	53%	−1,922,683 EUR	5
Spain	25,065,029 EUR	−1,058,434 EUR	0 EUR	0 EUR	−17,494,652 EUR	−18,553,086 EUR	159%	−90,017,925 EUR	3
Sweden	2,795,997 EUR	−131,605 EUR	0 EUR	0 EUR	−3,669,250 EUR	−3,800,855 EUR	383%	−24,579,274 EUR	2
Total EU27	354,813,518 EUR	−18,075,228 EUR	0 EUR	0 EUR	−189,710,489 EUR	−207,785,717 EUR	112%	−874,968,995 EUR	4
United Kingdom	30,925,733 EUR	−1,877,099 EUR	0 EUR	0 EUR	−19,204,524 EUR	−21,081,623 EUR	145%	−103,896,007 EUR	3
Total EU27 + UK	385,739,250 EUR	−19,952,327 EUR	0 EUR	0 EUR	−208,915,013 EUR	−228,867,340 EUR	114%	−978,865,002 EUR	4

**Table A5:** Economic impact after the full implementation of medication traceability system (in oncology) in all hospitals of each nation.

Country	Med, Traceability System (Oncology) Investment	Med, Traceability System (Oncology) HR Efficiency Savings	Med, Traceability System (Oncology) Wastage Reduction Savings	Med, Traceability System (Oncology) Inventory Reduction Savings	Med, Traceability System (Oncology) MAE Reduction Savings (Indirect)	Med, Traceability System (Oncology) Total Savings	Medication Traceability System ROI	Medication Traceability System NPV	Medication Traceability System Payback Time
Austria	7,139,199 EUR	−5,401,104 EUR	−921,596 EUR	0 EUR	−8,543,333 EUR	−14,866,032 EUR	518%	−99,450,091 EUR	2
Belgium	7,321,836 EUR	−5,195,135 EUR	−847,750 EUR	0 EUR	−7,743,082 EUR	−13,785,966 EUR	449%	−86,286,272 EUR	2
Bulgaria	5,711,427 EUR	−1,228,759 EUR	−602,398 EUR	0 EUR	−2,073,227 EUR	−3,904,384 EUR	107%	−17,017,091 EUR	3
Croatia	1,920,825 EUR	−450,644 EUR	−186,386 EUR	0 EUR	−907,066 EUR	−1,544,097 EUR	139%	−7,158,786 EUR	3
Cyprus	201,825 EUR	−48,504 EUR	−22,326 EUR	0 EUR	−225,455 EUR	−296,285 EUR	345%	−1,934,663 EUR	2
Czechia	7,125,032 EUR	−2,394,563 EUR	−899,189 EUR	0 EUR	−4,492,030 EUR	−7,785,781 EUR	231%	−45,779,342 EUR	2
Denmark	2,643,838 EUR	−2,107,660 EUR	−322,358 EUR	0 EUR	−2,932,290 EUR	−5,362,307 EUR	511%	−37,160,975 EUR	2
Estonia	946,907 EUR	−279,381 EUR	−77,448 EUR	0 EUR	−601,748 EUR	−958,578 EUR	198%	−4,976,153 EUR	2
Finland	2,188,886 EUR	−1,488,837 EUR	−222,928 EUR	0 EUR	−2,138,784 EUR	−3,850,549 EUR	437%	−26,822,861 EUR	2
France	65,922,887 EUR	−39,445,908 EUR	−7,220,223 EUR	0 EUR	−59,013,622 EUR	−105,679,754 EUR	389%	−719,754,013 EUR	2
Germany	77,699,282 EUR	−51,547,657 EUR	−10,713,028 EUR	0 EUR	−79,722,686 EUR	−141,983,371 EUR	447%	−946,016,662 EUR	2
Greece	2,920,845 EUR	−1,257,516 EUR	−310,071 EUR	0 EUR	−2,488,462 EUR	−4,056,050 EUR	317%	−25,267,350 EUR	2
Hungary	5,487,917 EUR	−1,274,387 EUR	−524,132 EUR	0 EUR	−2,590,023 EUR	−4,388,542 EUR	126%	−17,448,880 EUR	3
Ireland	3,607,810 EUR	−2,508,968 EUR	−511,685 EUR	0 EUR	−4,095,816 EUR	−7,116,469 EUR	493%	−48,759,946 EUR	2
Italy	11,085,190 EUR	−5,858,096 EUR	−1,537,276 EUR	0 EUR	−9,378,088 EUR	−16,773,460 EUR	351%	−105,306,173 EUR	2
Latvia	1,554,474 EUR	−258,706 EUR	−128,192 EUR	0 EUR	−782,684 EUR	−1,169,582 EUR	118%	−4,771,812 EUR	3
Lithuania	2,134,328 EUR	−356,139 EUR	−176,011 EUR	0 EUR	−1,031,684 EUR	−1,563,834 EUR	118%	−6,790,489 EUR	3
Luxembourg	197,703 EUR	−199,553 EUR	−23,673 EUR	0 EUR	−554,658 EUR	−777,885 EUR	1106%	−6,174,540 EUR	1
Malta	111,930 EUR	−54,295 EUR	−12,486 EUR	0 EUR	−97,758 EUR	−164,539 EUR	344%	−1,061,911 EUR	2
Netherlands	4,261,496 EUR	−3,150,739 EUR	−506,865 EUR	0 EUR	−4,987,577 EUR	−8,645,181 EUR	512%	−60,263,820 EUR	2
Poland	26,841,735 EUR	−7,151,569 EUR	−2,177,559 EUR	0 EUR	−12,722,500 EUR	−22,051,628 EUR	132%	−88,927,539 EUR	3
Portugal	6,561,246 EUR	−2,333,888 EUR	−741,064 EUR	0 EUR	−4,155,910 EUR	−7,230,861 EUR	231%	−41,606,148 EUR	2
Romania	13,378,190 EUR	−2,684,282 EUR	−1,363,600 EUR	0 EUR	−4,568,559 EUR	−8,616,440 EUR	85%	−29,240,266 EUR	4
Slovakia	3,334,065 EUR	−1,197,215 EUR	−325,570 EUR	0 EUR	−2,032,401 EUR	−3,555,186 EUR	208%	−17,895,938 EUR	2
Slovenia	1,168,362 EUR	−296,908 EUR	−116,159 EUR	0 EUR	−915,668 EUR	−1,328,736 EUR	237%	−7,417,004 EUR	2
Spain	9,207,562 EUR	−4,406,021 EUR	−1,112,370 EUR	0 EUR	−7,377,337 EUR	−12,895,728 EUR	314%	−77,234,481 EUR	2
Sweden	3,746,636 EUR	−1,828,773 EUR	−390,679 EUR	0 EUR	−3,946,595 EUR	−6,166,047 EUR	393%	−40,069,529 EUR	2
Total EU27	274,421,434 EUR	−144,405,207 EUR	−31,993,020 EUR	0 EUR	−230,119,044 EUR	−406,517,271 EUR	350%	−2,570,592,736 EUR	2
United Kingdom	29,688,703 EUR	−17,825,418 EUR	−4,036,774 EUR	0 EUR	−32,543,630 EUR	−54,405,822 EUR	453%	−371,331,564 EUR	2
Total EU27 + UK	304,110,137 EUR	−162,230,625 EUR	−36,029,794 EUR	0 EUR	−262,662,674 EUR	−460,923,093 EUR	360%	−2,941,924,300 EUR	2

## Appendix B

In the following appendix, we report the full list of cost items and country-specific input parameters used in the model. These include salary levels, treatment volumes, inflation rates, and cost benchmarks for hospital care and automation technologies. This information enhances transparency and supports the reproducibility and contextual interpretation of the results, especially for decision-makers evaluating technology adoption at the national level.

1. Human Resources
  - Pharmacist average hourly wage (EUR): country-specific, from SalaryExpert, OECD, or national statistics.
  - Nurse average hourly wage (EUR): used for technologies impacting preparation/administration phases.
  - Technical/logistics staff wage (EUR): relevant for inventory/transport automation.
  - Time saved per activity: estimated via the literature (% reduction per dose/patient) and expert validation (Table 1).
2. Hospital Activity and Size
  - Total number of hospital beds per country.
  - Annual number of oncology inpatients: used for technologies affecting cancer drug preparation.
  - Baseline operational volumes: modeled on an average 561-bed hospital and scaled nationally.
3. Medication-Related Costs
  - Drug wastage and expiry rates: estimated per dose or unit (literature-based).
  - Cost per wasted drug unit: derived from national pharma spending (e.g., OSMED).
  - Avoidable costs from Medication Errors (MEs):
  - Based on extended length of stay due to adverse events.
  - Valued using national cost per hospital day.
  - Proportional drug cost adjustment factor: based on cross-country comparison of pharma prices (e.g., Standards och Läkemedelsförmånsverket report, Sweden).
4. Inventory Holding and Optimization
  - Baseline stock value: estimated from hospital drug expenditure per bed.
  - Avoidable inventory cost: annual holding cost rate (4.65%) applied to optimized stock.
  - Technologies considered: inventory robots, ADCs.
5. Capital Investment and Technology Data
  - Technology acquisition prices: based on supplier benchmarks and market intelligence.
  - Technology penetration rates (baseline): obtained from ECAMET study and used to estimate marginal savings.
  - Discount rate (national inflation rate): used to discount future savings and evaluate NPV scenarios.
6. Cost of Hospital Care.
  - Cost per ordinary inpatient day: national DRG/tariff-based or benchmarked via WHO/OECD data.
  - Cost per ICU day: where relevant, e.g., to value MAEs with severe clinical consequences.
7. Economic Conversion and Standardization
  - Currency exchange rates: national-to-euro conversion using average annual rates.
  - National inflation rates: used for sensitivity scenarios on discounting.
