# Correction: Orsini et al. The Impact of Automation and Digitalization in Hospital Medication Management: Economic Analysis in the European Countries. *Healthcare* 2025, *13*, 1604

**DOI:** 10.3390/healthcare14172769

**Published:** 2026-09-01

**Authors:** Federico Filippo Orsini, Daniele Bellavia, Fabrizio Schettini, Emanuela Foglia

**Affiliations:** Healthcare Datascience LAB, LIUC—Università Carlo Cattaneo, 21053 Castellanza, Italy; forsini@liuc.it (F.F.O.); dbellavia@liuc.it (D.B.); efoglia@liuc.it (E.F.)

**Data Correction**

There was an error in the original publication [1]. One of the input parameters used in the economic model, namely the purchase price of one of the technologies included in the analysis, was wrong.

A correction has been made to the Results part of the Abstract, Table 1, the whole Results section, the first paragraph of the Discussion section, and the second paragraph of the Conclusions section, Tables A2 and A3.

**The Results part of the Abstract:**

Results: The analysis estimated a total European investment of €2.47 billion, with an average PBT of 3.46 years and annual savings of 1.96 billion. The ROI averaged 206%, and the total NPV was 10.68 billion. The major saving driver was the reduction in Medication Administration Errors that has an impact of 37.2% on the total savings. Payback times ranged from 3 years in high-GDP countries to 4 years in lower-GDP nations.

**Table 1:** Input parameters for economic evaluation of the technologies’ impact on an average European hospital.

Technology	Number of Medications Managed/Stocked in an Average European Hospital	Percentage of Expired or Wasted Drug/Number of Preparations	Percentage of Wasted Drugs’ Reduction Thanks to Automation	% Reduction in Product Stock Thanks to Automation	Number of Medications’ Errors Without Automation Technologies in a Year	Percentage of Medication Error Reduction Thanks to Automation	Professional	Number of Hours Dedicated to the Specific Activities Without Automation	Reduced Process Time Through Automation (% of Hours)
Inventory Robots	1,563,133	0.46% [10]	−100.00% [11]	−26.40% [12]	128.96 [10]	−16% [10]	Technicians	7769.45 [10]	−31.40% [10]
Unit Dose System	545,143	0.55% [11]	−100.00% [11]	0.00% (Assumption)	77.79 [13]	−53% [13]	Nurses	20,640.59 [10]	−5.84% [14]
							Technicians	13,881.92 [10]	−10.00% [10]
Automated Dispensing Cabinets	2,850,352	0.55% [11]	−100.00% (Assumption)	−60.58% Elaboration from [12]	954.91 Elaboration from [15]	−53% [16]	Nurses	499.16 [10]	−80.00% [10]
							Pharmacists	1992.81 [10]	−50.00% [17]
							Technicians	1996.63 [10]	15.00% [18]
Smart pumps with DERS	26,690	-	-	-	12.82 Hypothesis: Baseline efficacy is equal to the one expressed in [19]	−100% (Assumption)	Nurses	394.37 [20]	−69.81% [20]
Med. Traceability System in Oncology	19,180	2.5153% (Elaboration from [21])	−100.00% (Assumption)	-	284.82 [22]	−100% (Assumption)	Pharmacists (preparation)	2878.90 [23]	−44.38% [23]
		0.0960% [21]	−21.10% Elaboration from [21]		139.25 [24]	−75% [25–27]	Nurses (administration)	1917.99 [28]	−88.61% [28]

**The Results section:**

Table 2 summarizes the average economic results for each technology at the hospital level based on a 561-bed model. The findings account for country-specific variation in technology penetration, labor costs, and pharmaceutical pricing. Among the five technologies analyzed, the oncology medication traceability system demonstrates the most favorable economic profile, with the lowest average investment (−€78,549), high annual savings (€111,783), and a Net Present Value (NPV) of €696,935 over ten years. The inventory robot, while requiring a higher investment (−€190,163), yields a high NPV overall (€780,692), driven by substantial reductions in wastage and stock inefficiencies. Automated Dispensing Cabinets (ADCs) generate the highest annual savings (€151,310), showing a very positive NPV (€790,730) due to their initial costs (−€189,762). The unit-dose distribution system (UDDS) and DERS for ICU smart pumps occupy an intermediate position, combining moderate investment with solid savings and ROI profiles. These results highlight the importance of balancing investment requirements with achievable savings and suggest that technologies with strong safety and traceability components (such as oncology-focused systems) can offer substantial long-term value even with modest capital requirements.

To complement the hospital-level analysis, Table 3 presents the aggregated economic impact of implementing each technology across all acute care hospitals in the EU27 + UK. The estimates are based on standardized 561-bed hospital models, scaled by country-specific hospital counts and contextual variables (e.g., labor costs, drug prices, and penetration rates). Table 3 summarizes the key economic results per technology, highlighting their distinct profiles. The oncology medication traceability system emerged as the most economically favorable, with a 360% ROI, which is the shortest payback time (2 years) and substantial savings in both direct and indirect cost categories. Inventory robots also performed well, achieving a 253% ROI and over €474 million in annual savings, primarily due to reductions in drug wastage and improved stock efficiency. Similarly, Automated Dispensing Cabinets (ADCs) generated the highest total annual savings (€592 million), with an initial investment of €674 million, resulting in a significant ROI of 217% and a short payback time (3 years). These findings support differentiated investment strategies, depending on available capital, hospital size, and national healthcare cost structures, and offer policymakers a comparative overview of return potential across technologies at the system level.

The sensitivity analysis explored the effects of varying two structural assumptions: the size of the hospital infrastructure (±20% around the baseline of 561 beds) and the discount rate (±20%), which directly affects the Net Present Value (NPV) of the investments. Varying hospital size influenced the total economic returns at the European level, with the overall NPV ranging from approximately €9.68 billion in the low-volume scenario to €10.83 billion in the high-volume case. This confirmed the presence of economies of scale: larger infrastructures can dilute fixed investments more efficiently and generate proportionally higher returns. Varying the discount rate had a similar impact. At the European level, reducing the discount rate by 20% increased the total NPV to approximately €10.89 billion, while increasing it by 20% reduced the NPV to €10.49 billion, a difference of nearly €399 million. Similar shifts were observed at the hospital level, although on a smaller scale. Across both sensitivity dimensions, the medication traceability system consistently delivered the strongest performance, maintaining high ROI and low payback time in all scenarios. Table 4 resumes the main results for sensitivity analysis.

**The first paragraph of the Discussion section:**

The results of this study reinforce the economic viability of adopting automation and digital technologies in hospital medication management across Europe. With an estimated investment of €2.47 billion and an average payback time (PBT) of 3.46 years, the financial sustainability of these technologies emerges clearly, especially in countries with high labor and drug costs. These findings are consistent with Bonnabry et al. (2022), who estimated a PBT of 3.8 years for similar technologies in a Swiss context, thereby validating our approach and estimates across different settings [2].

**The second paragraph of the Conclusions section:**

Our findings confirm the economic sustainability of automation, with a total estimated investment of €2.47 billion and an average payback time of 3.46 years across EU27 + UK. Even under varying economic assumptions—such as changes in hospital size or inflation rates—the model remains robust, highlighting the resilience of these investments over time and across contexts.

**Table A2:** Economic impact after the full implementation of UDDS in all hospitals of each nation.

Country	Unit Dose System Investment	Unit Dose System HR Efficiency Savings	Unit Dose System Wastage Reduction Savings	Unit Dose System Inventory Reduction Savings	Unit Dose System MAE Reduction Savings (Indirect)	Unit Dose System Total Savings	Unit Dose System ROI	Unit Dose System NPV	Unit Dose System Payback Time
Austria	15,245,165 €	−4,751,008 €	−3,178,521 €	0 €	−450,934 €	−8,380,463 €	138%	−38,766,533 €	3
Belgium	2,440,612 €	−711,227 €	−456,403 €	0 €	−64,749 €	−1,232,380 €	113%	−5,009,576 €	4
Bulgaria	19,038,089 €	−1,133,957 €	−3,243,127 €	0 €	−460,100 €	−4,837,184 €	13%	−4,777,031 €	8
Croatia	6,402,752 €	−546,183 €	−1,003,449 €	0 €	−142,358 €	−1,691,990 €	14%	−1,694,651 €	8
Cyprus	828,924 €	−72,944 €	−148,096 €	0 €	−21,010 €	−242,050 €	30%	−472,765 €	7
Czechia	15,214,912 €	−3,928,253 €	−3,101,241 €	0 €	−439,971 €	−7,469,465 €	119%	−34,167,764 €	4
Denmark	3,946,027 €	−1,314,620 €	−777,079 €	0 €	−110,244 €	−2,201,942 €	147%	−10,845,899 €	3
Estonia	1,527,269 €	−163,943 €	−201,754 €	0 €	−28,623 €	−394,320 €	10%	−290,087 €	9
Finland	3,266,994 €	−899,022 €	−537,393 €	0 €	−76,239 €	−1,512,654 €	108%	−6,732,907 €	4
France	31,157,584 €	−7,201,779 €	−5,511,632 €	0 €	−781,931 €	−13,495,342 €	95%	−56,242,260 €	4
Germany	165,920,343 €	−42,755,678 €	−36,948,506 €	0 €	−5,241,853 €	−84,946,037 €	124%	−383,683,750 €	4
Greece	11,996,330 €	−1,736,215 €	−2,056,854 €	0 €	−291,804 €	−4,084,873 €	50%	−11,096,804 €	6
Hungary	18,293,058 €	−1,388,291 €	−2,821,767 €	0 €	−400,322 €	−4,610,379 €	2%	−681,393 €	10
Ireland	6,013,017 €	−1,550,142 €	−1,377,378 €	0 €	−195,407 €	−3,122,927 €	129%	−14,489,059 €	4
Italy	45,528,458 €	−9,601,665 €	−10,197,496 €	0 €	−1,446,710 €	−21,245,872 €	103%	−87,014,711 €	4
Latvia	2,507,216 €	−155,982 €	−333,943 €	0 €	−47,376 €	−537,301 €	−10%	463,170 €	Pay Back time is over 10 years
Lithuania	3,442,464 €	−214,805 €	−458,512 €	0 €	−65,049 €	−738,365 €	−7%	439,693 €	Pay Back time is over 10 years
Luxembourg	0 €	0 €	0 €	0 €	0 €	0 €	Penetration rate 100%	Penetration rate 100%	Penetration rate 100%
Malta	459,711 €	−81,305 €	−82,829 €	0 €	−11,751 €	−175,885 €	70%	−601,161 €	5
The Netherlands	0 €	0 €	0 €	0 €	0 €	0 €	Penetration rate 100%	Penetration rate 100%	Penetration rate 100%
Poland	43,293,122 €	−4,045,104 €	−5,672,575 €	0 €	−804,763 €	−10,522,442 €	−2%	1,375,267 €	Pay Back time is over 10 years
Portugal	1,198,401 €	−175,075 €	−218,612 €	0 €	−31,014 €	−424,700 €	56%	−1,258,320 €	5
Romania	44,593,967 €	−3,111,871 €	−7,341,210 €	0 €	−1,041,491 €	−11,494,572 €	7%	−5,286,506 €	9
Slovakia	11,113,550 €	−1,477,387 €	−1,752,772 €	0 €	−248,664 €	−3,478,824 €	30%	−6,029,583 €	7
Slovenia	3,894,540 €	−725,357 €	−625,367 €	0 €	−88,720 €	−1,439,444 €	59%	−4,260,977 €	5
Spain	0 €	0 €	0 €	0 €	0 €	0 €	Penetration rate 100%	Penetration rate 100%	Penetration rate 100%
Sweden	5,591,994 €	−1,538,227 €	−941,775 €	0 €	−133,609 €	−2,613,610 €	105%	−10,898,491 €	4
TOTAL EU27	462,914,499 €	−89,280,038 €	−88,988,291 €	0 €	−12,624,694 €	−190,893,022 €	84%	−682,022,097 €	5
United Kingdom	44,670,503 €	−11,452,446 €	−9,809,933 €	0 €	−1,391,727 €	−22,654,107 €	125%	−104,763,161 €	4
TOTAL EU27 + UK	507,585,002 €	−100,732,484 €	−98,798,224 €	0 €	−14,016,421 €	−213,547,129 €	88%	−786,785,258 €	4

**Table A3:** Economic impact after the full implementation of ADCs in all hospitals of each nation.

Country	Automated Dispensing Cabinets Investment	Automated Dispensing Cabinets HR Efficiency Savings	Automated Dispensing Cabinets Wastage Reduction Savings	Automated Dispensing Cabinets Inventory Reduction Savings	Automated Dispensing Cabinets MAE Reduction Savings (Indirect)	Automated Dispensing Cabinets Total Savings	ADC ROI	ADC NPV	ADC Payback Time
Austria	16,282,856 €	−3,330,725 €	−4,771,350 €	−1,889,720 €	−6,232,230 €	−16,224,025 €	251%	−92,577,587 €	2
Belgium	12,301,257 €	−2,396,951 €	−3,233,084 €	−1,280,481 €	−4,222,980 €	−11,133,496 €	212%	−57,922,269 €	2
Bulgaria	14,991,781 €	−1,084,398 €	−3,589,316 €	−1,421,569 €	−4,688,283 €	−10,783,566 €	160%	−55,837,206 €	3
Croatia	5,041,927 €	−341,090 €	−1,110,562 €	−439,844 €	−1,450,591 €	−3,342,087 €	133%	−15,245,293 €	3
Cyprus	1,059,986 €	−75,748 €	−266,163 €	−105,415 €	−347,656 €	−794,983 €	171%	−4,221,961 €	3
Czechia	16,250,543 €	−1,074,645 €	−4,655,344 €	−1,843,775 €	−6,080,705 €	−13,654,469 €	203%	−77,078,710 €	3
Denmark	4,601,746 €	−986,488 €	−1,273,636 €	−504,431 €	−1,663,595 €	−4,428,151 €	245%	−26,049,141 €	2
Estonia	2,075,385 €	−179,318 €	−385,321 €	−152,609 €	−503,298 €	−1,220,546 €	105%	−4,885,730 €	4
Finland	3,809,877 €	−729,463 €	−880,790 €	−348,842 €	−1,150,468 €	−3,109,561 €	197%	−17,650,917 €	3
France	101,615,103 €	−17,200,629 €	−25,263,440 €	−10,005,728 €	−32,998,533 €	−85,468,330 €	206%	−492,450,182 €	3
Germany	177,214,023 €	−33,049,912 €	−55,464,245 €	−21,966,927 €	−72,446,139 €	−182,927,223 €	267%	−1,085,169,342 €	2
Greece	15,175,357 €	−1,925,701 €	−3,656,889 €	−1,448,332 €	−4,776,546 €	−11,807,468 €	177%	−61,895,349 €	3
Hungary	14,405,097 €	−1,002,977 €	−3,122,977 €	−1,236,872 €	−4,079,161 €	−9,441,988 €	119%	−36,463,685 €	3
Ireland	4,278,637 €	−888,126 €	−1,377,476 €	−545,557 €	−1,799,228 €	−4,610,386 €	285%	−28,129,756 €	2
Italy	57,593,499 €	−8,405,259 €	−18,130,170 €	−7,180,556 €	−23,681,217 €	−57,397,201 €	253%	−331,708,499 €	2
Latvia	3,407,023 €	−186,043 €	−637,784 €	−252,598 €	−833,060 €	−1,909,485 €	92%	−6,893,601 €	4
Lithuania	4,677,919 €	−255,744 €	−875,692 €	−346,823 €	−1,143,810 €	−2,622,069 €	98%	−10,405,360 €	4
Luxembourg	703,389 €	−211,861 €	−191,189 €	−75,721 €	−249,727 €	−728,498 €	278%	−4,639,408 €	2
Malta	581,535 €	−82,032 €	−147,261 €	−58,324 €	−192,349 €	−479,966 €	196%	−2,649,681 €	3
The Netherlands	15,161,602 €	−2,923,760 €	−4,093,503 €	−1,621,255 €	−5,346,840 €	−13,985,357 €	231%	−81,343,488 €	2
Poland	58,830,448 €	−4,622,264 €	−10,833,812 €	−4,290,792 €	−14,150,880 €	−33,897,748 €	92%	−114,815,864 €	4
Portugal	11,201,059 €	−1,118,407 €	−2,871,759 €	−1,137,376 €	−3,751,027 €	−8,878,570 €	183%	−47,342,537 €	3
Romania	35,116,077 €	−2,202,736 €	−8,124,849 €	−3,217,892 €	−10,612,493 €	−24,157,970 €	133%	−101,880,329 €	3
Slovakia	8,751,504 €	−881,563 €	−1,939,872 €	−768,297 €	−2,533,817 €	−6,123,550 €	139%	−26,516,457 €	3
Slovenia	3,066,805 €	−167,032 €	−692,122 €	−274,119 €	−904,034 €	−2,037,306 €	133%	−9,242,138 €	3
Spain	31,151,171 €	−4,416,519 €	−8,542,769 €	−3,383,412 €	−11,158,371 €	−27,501,071 €	209%	−146,961,184 €	3
Sweden	6,521,227 €	−770,674 €	−1,543,574 €	−611,341 €	−2,016,181 €	−4,941,770 €	169%	−25,340,734 €	3
TOTAL EU27	625,866,834 €	−90,510,064 €	−167,674,950 €	−66,408,609 €	−219,013,218 €	−543,606,841 €	214%	−2,965,316,405 €	3
United Kingdom	48,901,315 €	−7,845,993 €	−15,093,277 €	−5,977,777 €	−19,714,496 €	−48,631,543 €	257%	−291,719,110 €	2
TOTAL EU27+UK	674,768,149 €	−98,356,058 €	−182,768,227 €	−72,386,386 €	−238,727,714 €	−592,238,384 €	217%	−3,257,035,515 €	3

The authors state that the scientific conclusions are unaffected. This correction was approved by the Academic Editor. The original publication has also been updated.

## Figures and Tables

**Table 2:** Economic impact of automated technologies on an average European hospital.

Hospital-Based Results			
Technology	Investment	Annual Savings	NPV
Inventory Robot	−190,163 €	140,488 €	780,692 €
UDDS	−151,171 €	55,301 €	186,550 €
ACDs	−189,762 €	151,310 €	790,730 €
DERS	−99,017 €	65,300 €	304,488 €
Med, Traceability System	−78,549 €	111,783 €	696,935 €
TOTAL	−708,662 €	524,183 €	2,759,396 €

**Table 3:** Economic impact of automated technologies on all European Healthcare Services.

Technology	Investment	HR Efficiency Savings	Wastage Reduction Savings	Inventory Reduction Savings	Mae Reduction Savings (Indirect Benefit)	Total Annual Savings	ROI	NPV	Payback Time
Inventory Robot	−600,130,638 €	105,401,590 €	298,558,225 €	61,611,921 €	8,807,528 €	474,379,264 €	253%	2,720,310,043 €	3.05
UDDS	−507,585,002 €	100,732,484 €	98,798,224 €	0 €	14,016,421 €	213,547,129 €	88%	786,785,258 €	4.93
ACDs	−674,768,149 €	98,356,058 €	182,768,227 €	72,386,386 €	238,727,714 €	592,238,384 €	217%	3,257,035,515 €	3.00
DERS	−385,739,250 €	19,952,327 €	0 €	0 €	208,915,013 €	228,867,340 €	114%	978,865,002 €	4.01
Med, Traceability System	−304,110,137 €	162,230,625 €	36,029,794 €	0 €	262,662,674 €	460,923,093 €	360%	2,941,924,300 €	2.33
TOTAL	−2,472,333,177 €	486,673,083 €	616,154,470 €	133,998,307 €	733,129,349 €	1,969,955,209 €	206%	10,684,920,118 €	3.46

**Table 4:** Main sensitivity analysis results.

Scenario	ROI	Total NPV (€)	Payback Time (Years)
Baseline	206%	10,684,920,118 €	3.46
Hospital size −20%	173%	9,678,342,613 €	4.04
Hospital size +20%	202%	10,832,339,432 €	3.61
Discount rate −20%	211%	10,889,349,589 €	3.23
Discount rate +20%	202%	10,490,602,158 €	3.50
